# RETRACTION: Salvianolic Acid B‐Induced MicroRNA‐152 Inhibits Liver Fibrosis by Attenuating DNMT1‐Mediated Patched1 Methylation

**DOI:** 10.1111/jcmm.71335

**Published:** 2026-08-24

## Abstract

**RETRACTION**: YuF. , LuZ. , ChenB. , WuX. , DongP. , and ZhengJ. , “Salvianolic Acid B‐Induced MicroRNA‐152 Inhibits Liver Fibrosis by Attenuating DNMT1‐Mediated Patched1 Methylation,” Journal of Cellular and Molecular Medicine 19, no. 11 (2015): 2617–2632, 10.1111/jcmm.12655.26257392 PMC4627567

The above article, published online on 10 August 2015 in Wiley Online Library (wileyonlinelibrary.com), has been retracted by agreement between the journal Editor‐in‐Chief, Stefan N. Constantinescu; the Foundation for Cellular and Molecular Medicine; and John Wiley & Sons Ltd. The retraction has been agreed due to concerns raised by third parties. Specifically, the GAPDH blot shown in Figure 1D was found to have been later published elsewhere by the same author group but presented in a different scientific context. Further investigation by the publisher identified additional concerns, namely that the GAPDH bands in Figures 3C and 5B had been previously published by a different author group in a different scientific context.

The clarification and materials provided by the authors were insufficient to address these concerns, and the authors did not supply the original data underlying the study upon request. Accordingly, the article has been retracted, as the editors consider its conclusions to be invalid. The authors have been informed of the retraction decision but were not available for final confirmation.

**RETRACTION**: F. Yu , Z. Lu , B. Chen , X. Wu , P. Dong , and J. Zheng , “Salvianolic Acid B‐Induced MicroRNA‐152 Inhibits Liver Fibrosis by Attenuating DNMT1‐Mediated Patched1 Methylation,” Journal of Cellular and Molecular Medicine 19, no. 11 (2015): 2617–2632, 10.1111/jcmm.12655.26257392 PMC4627567

The above article, published online on 10 August 2015 in Wiley Online Library (wileyonlinelibrary.com), has been retracted by agreement between the journal Editor‐in‐Chief, Stefan N. Constantinescu; the Foundation for Cellular and Molecular Medicine; and John Wiley & Sons Ltd. The retraction has been agreed due to concerns raised by third parties. Specifically, the GAPDH blot shown in Figure 1D was found to have been later published elsewhere by the same author group but presented in a different scientific context. Further investigation by the publisher identified additional concerns, namely that the GAPDH bands in Figures 3C and 5B had been previously published by a different author group in a different scientific context.

The clarification and materials provided by the authors were insufficient to address these concerns, and the authors did not supply the original data underlying the study upon request. Accordingly, the article has been retracted, as the editors consider its conclusions to be invalid. The authors have been informed of the retraction decision but were not available for final confirmation.

